# Development of a High-Temperature Co-Fe-Si-B Amorphous Wire Fluxgate Magnetometer for Downhole Attitude Measurement in MWD Systems at Temperatures up to 175 °C

**DOI:** 10.3390/s25195972

**Published:** 2025-09-26

**Authors:** Bin Yan, Wanhua Zhu, Xin Zhuang, Zheng Lu, Guangyou Fang

**Affiliations:** 1 Aerospace Information Research Institute, Chinese Academy of Sciences, Beijing 100190, China; whzhu@mail.ie.ac.cn (W.Z.); zhuangxin@aircas.ac.cn (X.Z.); gyfang@mail.ie.ac.cn (G.F.); 2 Key Laboratory of Electromagnetic Radiation and Sensing Technology, Chinese Academy of Sciences, Beijing 100190, China; 3 Institute of Remote Sensing Satellite, China Academy of Space Technology, Beijing 100094, China; apps2006@163.com

**Keywords:** fluxgate sensor, measurement while drilling, high temperature, low noise

## Abstract

Measurement While Drilling (MWD) systems require high-precision triaxial magnetometers for real-time downhole attitude sensing, yet conventional fluxgates fail to meet the stringent size, noise, bandwidth, and temperature demands of deep reservoirs (>175 °C). To bridge this gap, we present a miniaturized triaxial fluxgate magnetometer (23 × 23 × 21 mm^3^) leveraging Co-Fe-Si-B amorphous wire cores—a material selected for its near-zero magnetostriction and tunable magnetic anisotropy. The sensor achieves breakthrough performance: a 300 Hz bandwidth combined with noise levels below 200 pT/√Hz at 1 Hz when operating at 175 °C while maintaining full functionality with the probe surviving temperatures exceeding 200 °C. This advancement paves the way for more accurate wellbore positioning and steering in high-temperature hydrocarbon and geothermal reservoirs.

## 1. Introduction

Borehole logging systems are critical for measuring geological parameters during drilling operations. Among these, Measurement While Drilling (MWD) systems enable real-time acquisition of drilling tool attitude (inclination, azimuth, tool face) by fusing triaxial accelerometer and magnetometer data. Fluxgate magnetometers dominate as the preferred magnetic sensor in commercial MWD systems (e.g., Schlumberger’s PowerDrive, Noble Drilling’s Well Direction) due to their DC-100 Hz bandwidth suitability, vector-field measurement capability, and compact form factor [1,2].

The fluxgate magnetometer, first conceptualized by H. Aschenbrenner and G. Goubauin the 1930s, has evolved through key innovations: F. Förster’s parallel-rod core design (1941) and the single-core, single-coil configuration [3,4,5]. Contemporary research in the UK, Ukraine, Germany, and the USA has further refined fluxgate technology, yielding low-noise sensors such as Bartington Mag-03 (<6 pT/√Hz @1 Hz) and LEMI’s LEMI-029 (<6 pT/√Hz @1 Hz) [6,7].

However, progress in high-temperature fluxgate sensors for downhole applications remains limited. Few commercial solutions tolerate the >175 °C temperatures typical of deep reservoirs. Bartington’s high-temperature Mag-610/Mag-614 (175 °C) and Mag-611 (215 °C) series represent the state of the art, yet their noise performance (~1nT/√Hz @1 Hz) falls short of precision attitude-sensing requirements in noisy MWD environments. Moreover, the manual does not provide the noise level of the sensor at high temperatures [8,9]. The pioneering work by Aimin Du’s team demonstrated a significant achievement in high-temperature stability with a ring-core fluxgate operational up to 220 °C using an Fe-based nanocrystalline core; however, the critical parameter of sensor noise performance was not characterized in their study [10]. Conversely, this study primarily addressed the design and validation of high-temperature digital circuitry, without detailing the development or performance characteristics of the sensor itself [11].

This gap underscores an urgent need for fluxgate sensors that concurrently achieve

1. High-temperature resilience (>150 °C);
2. Ultra-small volume;
3. Relatively low noise and robustness in high-vibration, high-pressure downhole settings.

In this work, we address these challenges by developing a novel high-temperature fluxgate magnetometer leveraging Co-Fe-Si-B amorphous wire cores. This material system offers exceptional thermal stability, near-zero magnetostriction, and tunable magnetic anisotropy, enabling superior signal-to-noise ratios at elevated temperatures. We present the design, fabrication, and characterization of a miniaturized triaxial fluxgate sensor (23 × 23 × 21 mm) optimized for downhole attitude measurement in MWD systems, demonstrating a 300 Hz bandwidth along with operational stability and noise performance of <200 pT/√Hz @1 Hz at 175 °C—matching its room-temperature baseline. Meanwhile, the fluxgate magnetometer maintains full functionality with the probe surviving temperatures exceeding 200 °C. This advancement paves the way for more accurate wellbore positioning and steering in high-temperature hydrocarbon and geothermal reservoirs.

To quantitatively illustrate the performance advantages of our sensor, a direct comparison with state-of-the-art commercial high-temperature fluxgate magnetometers is presented in Table 1.

## 2. Sensor Probe Design

The fluxgate sensor probe in this work consists mainly of three components: a magnetic core, an excitation coil, and a pick-up coil. In fact, the coils are made of enameled copper wire and can operate over a wide range of temperatures. Therefore, the key point is to identify a magnetic material with high temperature resistance as the cores in the probe [12,13,14].

In this respect, amorphous material is more advantageous than permalloy material. Amorphous materials are understood to be more sensitive to heat than permalloy materials since amorphous materials do not have crystalized structures. Their magnetic properties can be easily changed by thermal treatments over around 150 °C. Permalloy, on the other hand, has a crystalized structure and is usually annealed at more than 1000 °C. Moreover, an amorphous wire core has better centrosymmetry than amorphous stripes. In recent years, amorphous wires with lower noise levels and fewer defects have been developed. This leads to better performance of the sensor probe. The CoFeSiB amorphous wire used in this work was provided by Ningbo Institute of Materials Technology & Engineering, Chinese Academy of Sciences, CNITECH, and its Curie temperature was more than 300 °C. Therefore, choosing amorphous wire as the core of a high-temperature fluxgate sensor is possible [15,16,17,18].

To study the features of the amorphous wire, we measured the inductance of the coil with the core inside under different excitation voltages or different testing temperatures. The dimensions of the core and coil are given in Table 2.

The fluxgate sensor that we designed is a parallel-mode fluxgate sensor. Therefore, the inductance Lc and resistance Rc of the excitation coil with the magnetic core inside were both measured from 40 Hz to 100 kHz by using a precision impedance analyzer (Agilent 4294a, San Jose, CA, USA) at room temperature. The measuring model in the precision impedance analyzer was Ls (Inductance)–R (Resistance).

The inductance (Ls) is determined by the permeability (μ_app_) of the core and correlates with the coil’s field-to-voltage conversion coefficient (SF). Based on Faraday’s law of electromagnetic induction, the relationship is

(1) $$L_{s}=\frac{N^{2}\mu_{0}\mu_{app}\times \pi \times (\frac{d}{2}{)}^{2}}{l}$$

(2) $$SF=\frac{e}{B}=-j2\pi f\mu_{app}NS$$

N is the number of coil turns; μ0 is the vacuum permeability; μapp represents the effective permeability of the magnetic core and represents the degree of magnetic concentration of the magnetic field; d is the diameter of the core; l is the length of the core; e is the inductive voltage of the coil; B is the magnetic induction intensity; f is the frequency; and S represents the area of the coil or magnetic core.

The resistance contributes to the overall noise of the sensor in the form of thermal noise. The Johnson–Nyquist noise is

(3) $$e_{R}\left(f\right)=\sqrt{4k_{B}T_{c}R}\;\;\;(V/\sqrt{Hz})$$

k_B_ is the Boltzmann constant (1.3806503 × 10^−23^ J/K) and Tc is the absolute temperature.

**Figure 1:**
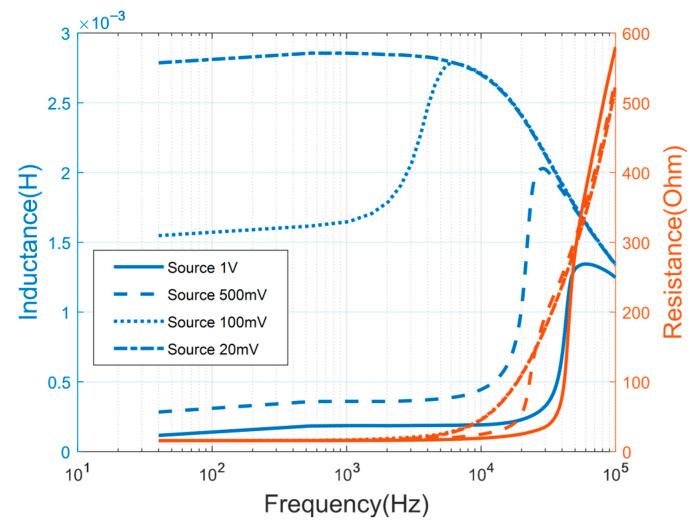
Inductance and resistance of the coil as a function of frequency (source 500 mV) The inductance is in blue lines and left axis, the resistance is in red lines and right axis.

Firstly, the impedance of the coil with a CoFeSiB core was measured at room temperature under different excitation voltages. The result is shown in Figure 1.

Obviously, the measured impedance values of the coil with the magnetic core vary with different excitation voltage amplitudes generated by the precision impedance analyzer. The observed decrease in coil inductance with increasing source voltage indicates a reduction in the effective magnetic permeability of the core, which aligns with characteristic changes in the magnetic hysteresis curve. This amplitude–frequency dependence can be primarily attributed to the skin effect, which becomes more pronounced at higher frequencies and leads to non-uniform magnetic flux distribution within the core material, thereby affecting the overall impedance characteristics. When the excitation voltage amplitude exceeds 20 mV, the magnetic core begins to enter the saturation region, further confirming the nonlinear magnetic behavior under strong driving conditions.

With an increase in the source amplitude, the frequency band range of magnetic core saturation also increases. This indicates that as the frequency increases, the magnetic field converging inside the magnetic core decreases. When the frequency increases to a certain extent, the magnetic core is no longer saturated under the outer field of the same amplitude. In other words, as the excitation frequency increases, the core needs a stronger excitation field to saturate. It is implied that the excitation voltage and the modulation and demodulation frequency need to be optimized as an ensemble for the sensor design. In this work, the voltage and the modulation and demodulation frequency were chosen to be 500 mV and 30 kHz.

Numerous studies have investigated the temperature-dependent magnetic properties of amorphous microwires, particularly focusing on the evolution of magnetic permeability under thermal influence [19]. For instance, research on Co-rich amorphous microwires has demonstrated that heat treatment near the crystallization onset temperature can significantly alter their micromagnetic structure, magnetostriction constant, and, consequently, soft magnetic properties.

**Figure 2:**
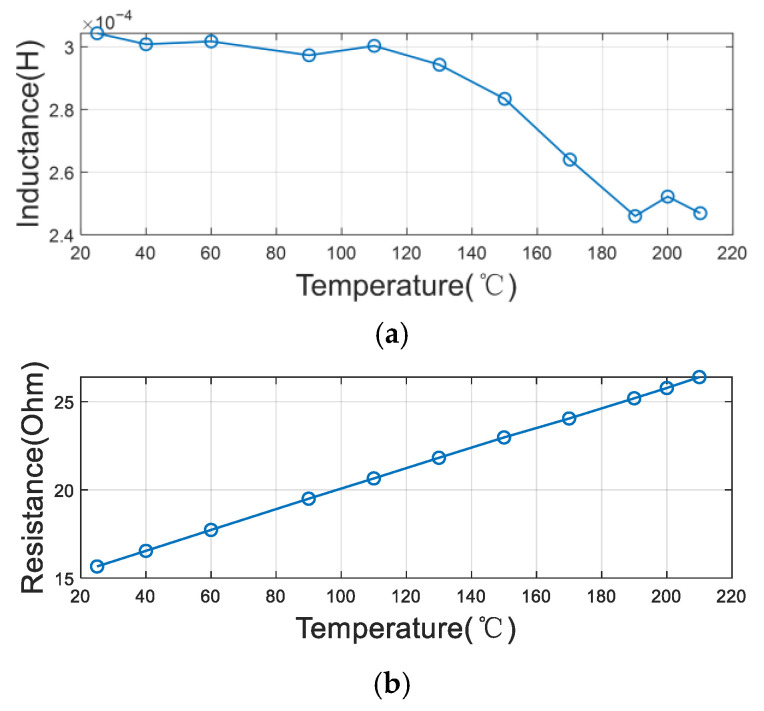
The variation in coil parameters with temperature at a frequency of 100 Hz: (**a**) inductance (L); (**b**) resistance (R).

**Figure 3:**
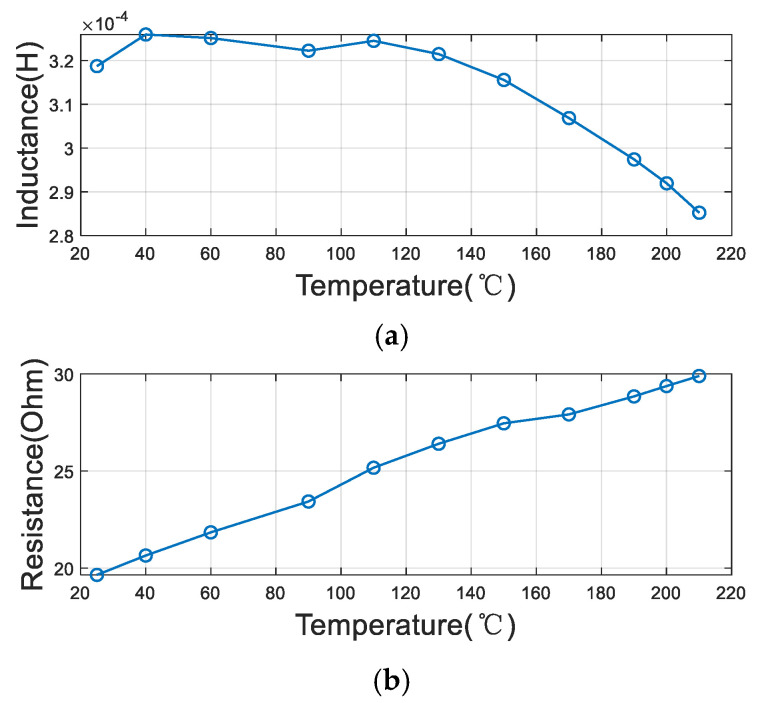
The variation in coil parameters with temperature at a frequency of 15 kHz: (**a**) inductance (L); (**b**) resistance (R).

**Figure 4:**
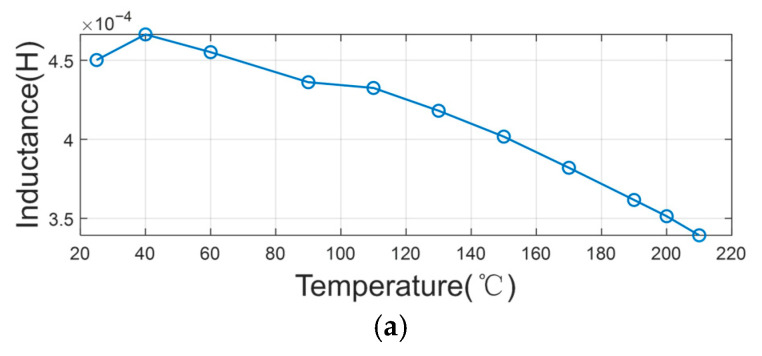

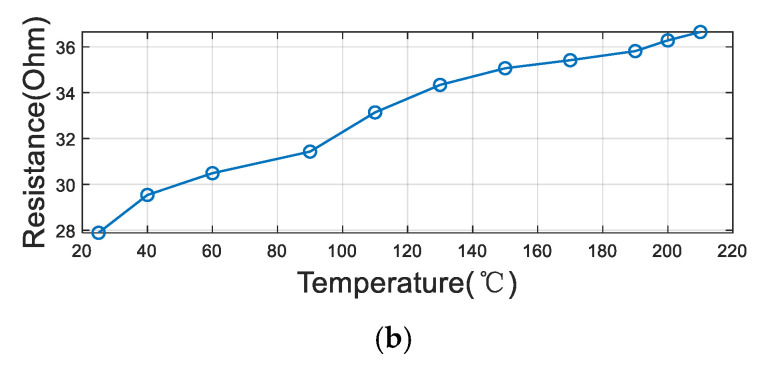
The variation in coil parameters with temperature at a frequency of 30 kHz: (**a**) inductance (L); (**b**) resistance (R).

In line with these investigations, we conducted systematic measurements on our Co-Fe-Si-B amorphous wire core. The core and coil were placed in a temperature-controlled chamber where the temperature was precisely varied from room temperature to 210 °C. The inductance and resistance values were measured across multiple frequencies (100 Hz, 15 kHz, and 30 kHz) within the 100 Hz to 100 kHz range. The impedance exhibited distinct variation patterns at different frequencies, with results comprehensively detailed in Figure 2, Figure 3 and Figure 4. These findings contribute to the understanding of thermal effects on amorphous magnetic materials under practical operating conditions.

From the results, it is observed that the inductance of the coil decreases and the resistance of the coil increases with an increase in temperature at the three frequency points. This reveals that the permeability of the core decreases and the thermal noise becomes greater when the temperature increases. Moreover, the magnitude of this change varies with different frequencies. On average, the value of the inductance decreases by a factor of 10% and the value of the resistance increases by a factor of 50%, which means that the noise voltage increases by about 22%.

## 3. Electronic Unit Design

**Figure 5:**
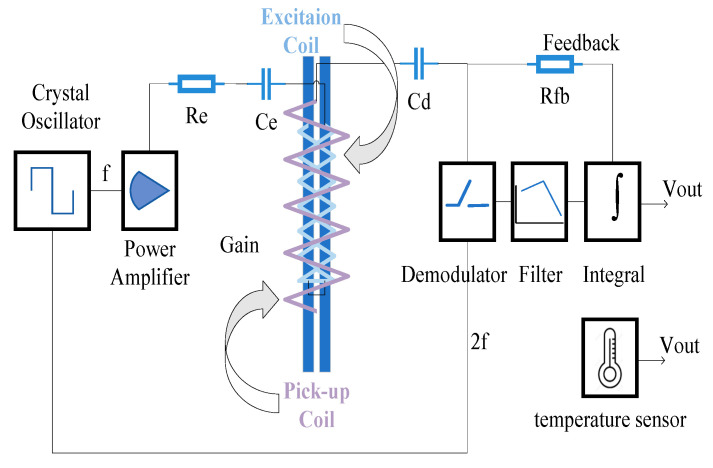
Function diagram of the fluxgate sensor.

For borehole logging applications, electronic units are required to operate reliably at high temperatures. At depths of 1000 m underground, ambient temperatures can exceed 100 °C. Therefore, the circuit design criterion focuses on maintaining stable signal modulation and demodulation functions over a wide temperature range. The signal processing circuit for the fluxgate sensor, shown schematically in Figure 5, consists of three main subsystems—power management, excitation circuitry, and detection circuitry—in addition to the previously mentioned high-temperature crystal oscillator, power amplifier demodulator, filter, and integrator [20,21,22,23,24,25].

The power management module provides regulated power to the entire electronic unit. The external power input is stabilized and filtered through this module to supply low-noise, regulated voltage to all components. The excitation circuitry generates periodic square-wave signals to drive the excitation coils and stimulate the magnetic core. The detection circuit demodulates the induced signals from the sensing coils, outputs the magnetic field signal, and connects to a feedback network coupled with feedback coils to adjust the sensor’s response.

All integrated circuits (ICs) in this system must maintain normal operation under high-temperature and high-vibration conditions. Regarding the theoretical aspects of circuit design, a detailed discussion has been provided in Reference 18 and thus will not be reiterated here. To address the specific application environment outlined in this work, we replaced conventional room-temperature components with high-temperature integrated circuits (ICs). This modification ensures long-term stable operation at 175 °C, meeting demanding downhole operational requirements.

We further integrated a temperature monitoring subsystem into the circuit using the AD590 sensor IC. This addition provides real-time thermal diagnostics essential for deployment in downhole environments. Future work will focus on implementing temperature compensation algorithms leveraging this sensor data to enhance measurement stability across extreme thermal gradients.

## 4. Realization

Based on the optimized magnetic core configuration with excellent high-temperature stability and the rigorously validated electronic design ensuring reliable signal processing under extreme conditions, we successfully developed a miniaturized fluxgate magnetometer capable of high-precision magnetic field measurement in continuous high-temperature environments. This system integrates the sensing probe and conditioning circuitry within the stringent size constraints of typical wellhead deployment scenarios, offering a practical solution for downhole magnetic monitoring. The final parameters of the fluxgate sensor are listed in Table 3.

The sensing probe and electronics unit are designed as separate modular components to meet the stringent spatial constraints and thermal management requirements of downhole deployment. The probe assembly is mounted inside the well casing, where it is directly exposed to high-temperature and high-pressure conditions. Its structural support is fabricated from Poly Ether Ether Ketone (PEEK), a high-performance thermoplastic material selected for its exceptional thermal stability, mechanical strength, and low magnetic permeability, ensuring minimal interference with magnetic measurements.

**Figure 6:**
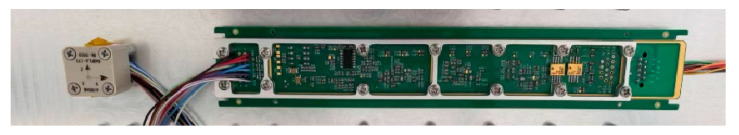
Appearance of the packaged fluxgate magnetometer.

The probe and the electronics unit are interconnected using a specialized high-temperature-resistant cable designed to maintain signal integrity and electrical insulation under extreme thermal cycling and mechanical stress. A photograph of the fully assembled fluxgate magnetometer, illustrating the integration of the probe and electronics within the compact form factor, is provided in Figure 6.

## 5. Experiments

**Figure 7:**
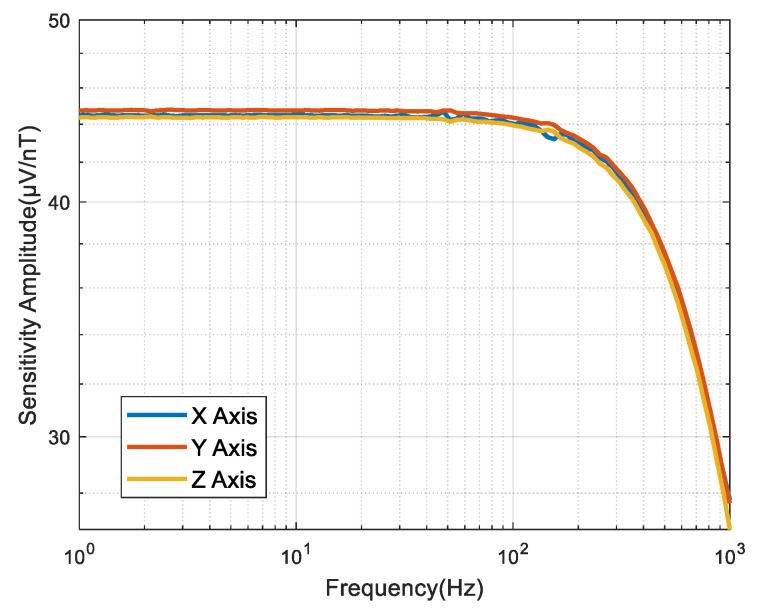
Sensitivity of the fluxgate magnetometer.

We used a Helmholtz coil (Bartington HC1, Witney, Oxon, UK) and a Dynamic Signal Analyzer (Agilent 35670A, San Jose, CA, USA) to test the performance of the fluxgate magnetometer [26,27,28]. The sensor achieved a measured sensitivity of 45 mV/μT with a 300 Hz bandwidth, as detailed in Figure 7.

**Figure 8:**
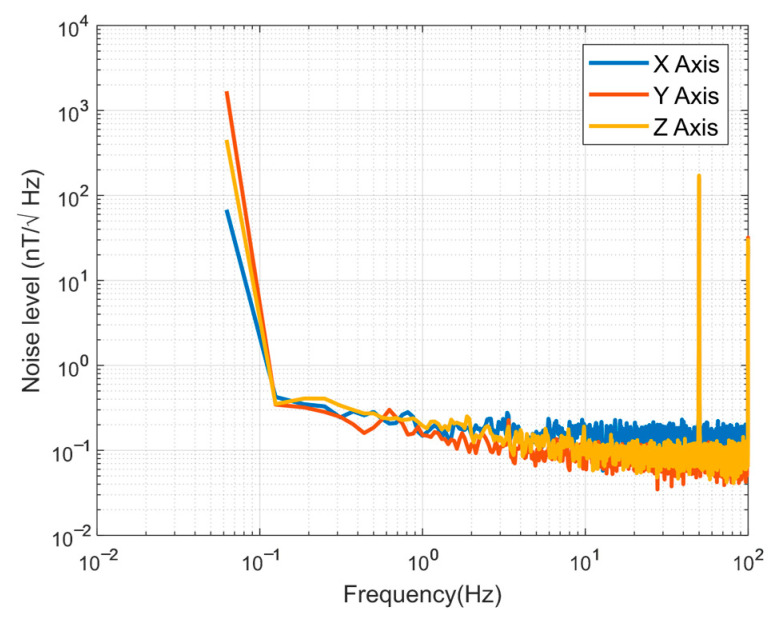
Noise level of the fluxgate sensor.

The equivalent input magnetic noise level of the sensor at 25 °C was measured by using an Agilent 35670A Dynamic Signal Analyzer, as shown in Figure 8. The noise values were about 200 pT/√Hz @1 Hz.

**Figure 9:**
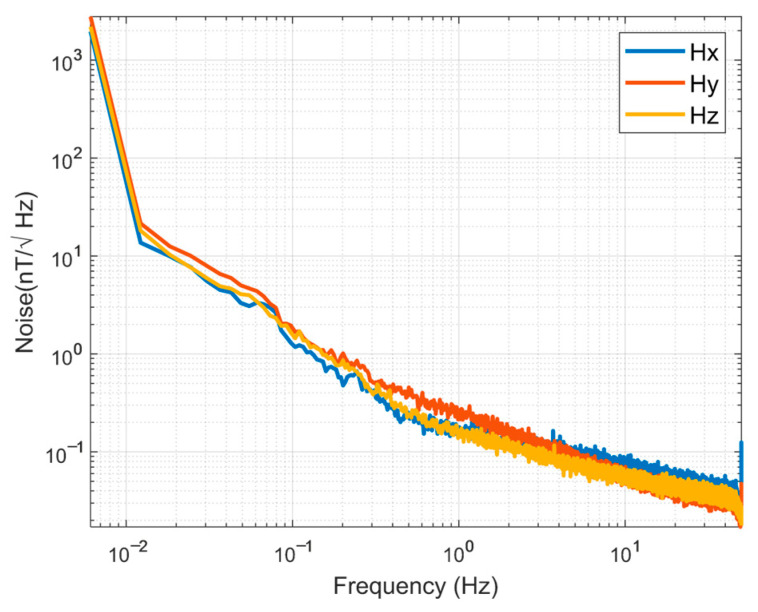
Noise level of the fluxgate sensor at 175 °C.

To validate the sensor’s noise performance under high-temperature conditions, the device was positioned within a magnetically shielded chamber. A closed-loop thermal oil circulation system delivered continuously heated oil into the chamber, elevating the ambient temperature until the sensor reached thermal equilibrium. This heat transfer methodology ensured that no additional electromagnetic interference was introduced during testing. Output signals were continuously recorded over extended durations (≥30 min) using a National Instruments data acquisition system. The acquired time-domain data was processed to calculate the magnetic noise power spectral density (PSD) via Welch’s method. As demonstrated in Figure 9, the resulting PSD confirmed stable noise characteristics across the target temperature range.

**Figure 10:**
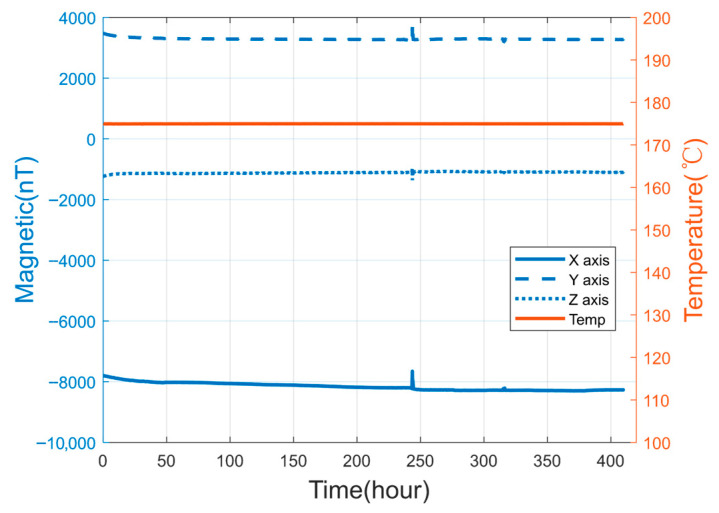
Results from a 400-h continuous high-temperature test.

As shown in Figure 10, the fluxgate sensor can operate normally at high temperatures, with a noise level of approximately 200 pT/√Hz @1 Hz; this is almost the same as that at room temperature, ensuring reliable performance in high-temperature downhole environments.

Furthermore, the sensor demonstrated robust long-term reliability by successfully completing a 400-h continuous high-temperature test and a random-vibration test exceeding 100 h at 20 g for each directional axis. As shown in Figure 10, the results revealed two notable phenomena: first, the environmental chamber provided certain magnetic shielding effects against the geomagnetic field; second, detectable output drift was observed during temperature variations, attributable to both operational influences from the chamber and inherent temperature drift of the sensor itself. These aspects represent important directions for our future research aimed at further enhancing thermal stability.

## 6. Conclusions

This work presented a miniaturized triaxial fluxgate magnetometer employing Co-Fe-Si-B amorphous wire cores for high-temperature downhole attitude measurement in MWD systems. The sensor achieves three critical advancements:

1. Unprecedented compactness (23 × 23 × 21 mm^3^ probe volume);
2. Thermally stable noise performance (<200 pT/√Hz @1 Hz at 175 °C, matching room-temperature baselines) with a 300 Hz bandwidth;
3. Extended thermal resilience (probe structural integrity maintained at >200 °C with full operation validated at 175 °C).

The sensor demonstrates downhole-grade robustness, withstanding 400 h of continuous bias-aging and >10 h of random vibration at 20 g (20 Hz–500 Hz) without performance degradation, confirming long-term reliability in extreme borehole environments.

Key innovations include the optimized 120 μm diameter Co_68.2_Fe_4.3_Si_12.5_B_15_ wire core enabling near-zero magnetostriction, precision solenoidal coils delivering 32 mV/nT sensitivity, and a high-temperature excitation system utilizing a dedicated HT-grade crystal oscillator for stable drive signals. The conditioning circuit implements radiation-hardened amplifiers, while Helmholtz coil calibration and magnetic shielding tests (Figure 10) confirm 300 Hz bandwidth stability and noise immunity across thermal gradients under simulated downhole conditions.

This sensor demonstrates 5× lower noise than state-of-the-art alternatives (e.g., Bartington Mag-611) at comparable temperatures while maintaining a wider 300 Hz bandwidth, enabling precise wellbore navigation in geothermal/ultra-deep reservoirs.

Future work will focus on two main directions: (1) integration with commercial MWD systems for field validation under real drilling conditions, and (2) the development of advanced temperature compensation algorithms using integrated temperature sensors and adaptive signal processing techniques to further enhance measurement accuracy in dynamic thermal environments.

## Figures and Tables

**Table 1:** Performance comparison between state-of-the-art commercial high-temperature fluxgate magnetometers and this work.

Parameters	Bartington Mag-610/614	Bartington Mag-611	This Work
Operating Temperature	175 °C	215 °C	175 °C
Noise @ 1 Hz	~1 nT/√Hz	~1 nT/√Hz	<200 pT/√Hz
Bandwidth	100 Hz	100 Hz	300 Hz

**Table 2:** Parameters of the core and coil.

Parameters	Values
Core length (l_core_)	17 mm
Core diameter (d_core_)	120 μm
Coil length (l_coil_)	15 mm
Coil turns (N)	900
Wire diameter of coil (d_coil_)	0.05 mm

**Table 3:** Optimum parameters of fluxgate sensor.

Parameter	Symbol	Value
Diameter of core	Φ_core_	120 μm
Length of core	L	17 mm
Wire diameter of excitation coil	Φ_exc_	0.05 mm
Turn number of excitation coil	N_exc_	900
Wire diameter of pick-up coil	Φ_pick-up_	0.08 mm
Turn number of pick-up coil	N_pick-up_	900
Feedback resistance	R_fb_	7 kΩ

## Data Availability

The data presented in this study are available on request from the corresponding author.
